# FOXO transcription factors protect against the diet-induced fatty liver disease

**DOI:** 10.1038/srep44597

**Published:** 2017-03-16

**Authors:** Xiaoyan Pan, Yang Zhang, Hyeong-Geug Kim, Suthat Liangpunsakul, X. Charlie Dong

**Affiliations:** 1 Department of Endocrinology, The First Affiliated Hospital, Wenzhou Medical University, Wenzhou, Zhejiang 325000, China; 2 Department of Biochemistry and Molecular Biology, Indiana University School of Medicine, Indianapolis, Indiana 46202, USA; 3 Division of Gastroenterology and Hepatology, Department of Medicine, Indiana University School of Medicine, Indianapolis, Indiana 46202, USA; 4 Roudebush Veterans Administration Medical Center, Indianapolis, Indiana 46202, USA

## Abstract

Forkhead O transcription factors (FOXOs) have been implicated in glucose and lipid homeostasis; however, the role of FOXOs in the development of nonalcoholic fatty liver disease (NAFLD) is not well understood. In this study, we designed experiments to examine the effects of two different diets—very high fat diet (HFD) and moderately high fat plus cholesterol diet (HFC)—on wildtype (WT) and liver-specific Foxo1/3/4 triple knockout mice (LTKO). Both diets induced severe hepatic steatosis in the LTKO mice as compared to WT controls. However, the HFC diet led to more severe liver injury and fibrosis compared to the HFD diet. At the molecular levels, hepatic Foxo1/3/4 deficiency triggered a significant increase in the expression of inflammatory and fibrotic genes including Emr1, Ccl2, Col1a1, Tgfb, Pdgfrb, and Timp1. Thus, our data suggest that FOXO transcription factors play a salutary role in the protection against the diet-induced fatty liver disease.

Forkhead O transcription factors (FOXOs) play a critical role in the integration of hormonal and nutritional signals for metabolic control^1^. As a downstream mediator of insulin signaling, the activity of FOXOs is modulated by serine/threonine phosphorylation catalyzed by Akt and other kinases. This insulin-mediated regulation has been implicated in the metabolic control during fasting and feeding cycles. In general, during feeding FOXOs are largely inhibited by the insulin-stimulated Akt whereas during fasting FOXOs are less phosphorylated and actively promote gluconeogenesis and lipolysis and inhibit glycolysis and lipogenesis^2^,^3^,^4^,^5^,^6^,^7^,^8^,^9^,^10^,^11^,^12^,^13^.

With regard to hepatic lipid metabolism, FOXOs play a key role in integrating multiple pathways. FOXOs have been shown to inhibit lipogenesis through suppression of genes such as sterol regulatory element binding protein 1c (SREBP-1c) and glucokinase^6^,^14^,^15^,^16^,^17^,^18^,^19^. FOXOs also interplay with sirtuin proteins, especially SIRT1 and SIRT6^6^,^7^,^20^,^21^,^22^,^23^,^24^,^25^. As sirtuins are NAD-dependent enzymes, FOXOs have been shown to modulate sirtuin activities through control of expression of nicotinamide phosphoribosyltransferase (NAMPT), the rate-limiting enzyme in the NAD biosynthesis^8^. Both SIRT1 and SIRT6 have been shown to inhibit lipogenesis and promote fatty acid oxidation in the liver^24^. FOXOs also promote hepatic lipolysis by upregulation of adipose triglyceride lipase (ATGL) that catalyzes the first step of lipolysis and downregulation of the G0/S1 switch 2 gene (G0S2), an inhibitor of ATGL^4^. In addition, FOXOs also increase breakdown of lipid droplets through activation of the autophagy pathway as autophagy related 14 (ATG14) has been shown to be a direct target of FOXOs^26^.

Both endogenous and exogenous factors can cause dysregulation of hepatic lipid homeostasis. One of the critical environmental factors is diet. It has been shown that diets containing excessive amount of fat, especially saturated fatty acids, remarkably increase the risk of obesity and nonalcoholic fatty liver disease (NAFLD)^27^,^28^,^29^,^30^. Additionally, dietary cholesterol has been attributed to the development of NAFLD^27^,^31^,^32^,^33^,^34^,^35^,^36^. We have recently reported that FOXOs regulate both total cholesterol and low-density lipoprotein cholesterol^7^,^25^. Generally, NAFLD is a progressive liver disease, beginning with simple steatosis, progressing to nonalcoholic steatohepatitis (NASH) and fibrosis, and in some cases ending up as cirrhosis and hepatocellular carcinoma^37^. Understanding the mechanism and pathogenesis leading to hepatic steatosis or NASH is of importance, as early interventions will likely either delay or reverse the disease progression.

To better understand the gene (notably FOXOs)-environment (different types of diets) interaction in the development of NAFLD, we set out to characterize wildtype (WT) and Foxo1/3/4 liver-specific triple knockout mice (LTKO) that were fed either regular chow, simply high-fat or moderately high-fat plus cholesterol diet.

## Results

### Dietary effects on food intake, body weight, liver, and adipose depot

**Figure 1:**
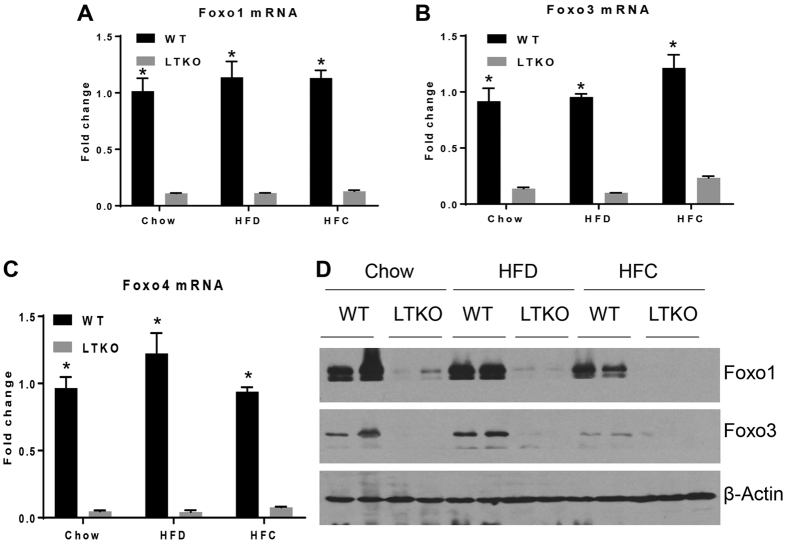
Generation of Foxo1/3/4 liver-specific knockout mice (LTKO). (**A**–**C**) Real-time PCR analysis of Foxo1, Foxo3, and Foxo4 mRNAs in the WT and LTKO mouse livers (n = 3), respectively *p < 0.05. (**D**) Western blot analysis of Foxo1 and Foxo3 proteins in WT and LTKO livers. The blots for the proteins shown are in the cropped format, and the full-length blots are presented in the Supplementary Figure 1. As Foxo4 protein is barely detectable in the mouse liver, Foxo4 Western blot data were not presented.

**Figure 2:**
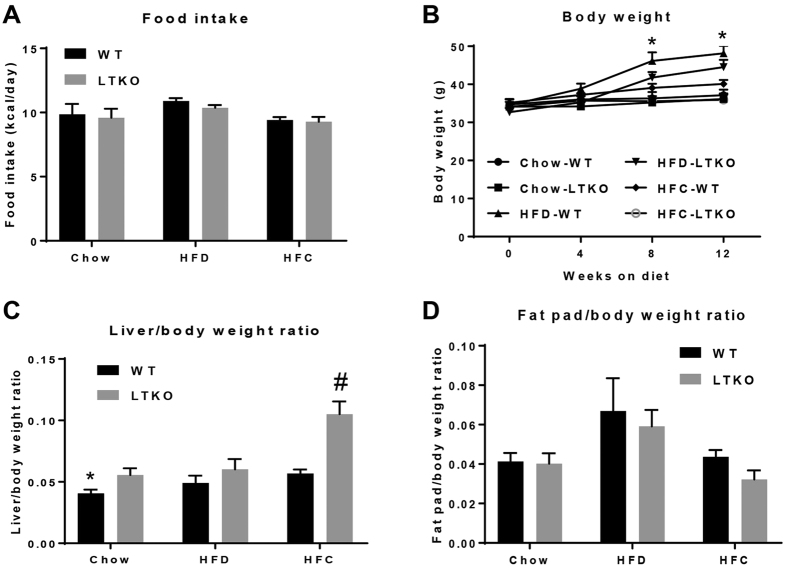
Animal food intake and body weight. (**A**) Food intake measurements. (**B**) Body weight measurements (n = 7, *p < 0.05 for Chow-WT vs. HFD-WT, Chow-LTKO vs. HFD-LTKO, HFC-WT vs. HFD-WT, HFC-LTKO vs. HFD-WT, and HFC-LTKO vs. HFD-LTKO). (**C**) Liver/body weight ratio (n = 7, *p < 0.05 for chow groups, ^#^*p* < 0.001 for HFC groups). (**D**) Perigonadal fat/body weight ratio in WT and LTKO mice that were fed chow, HFD, or HFC diet (n = 7).

Foxo1/3/4 gene knockout in the liver was confirmed by real-time PCR and immunoblotting (Fig. 1A–D). It was also noteworthy that both Foxo1 and Foxo3 genes were downregulated at both mRNA and protein levels in the liver of WT mice fed the HFC diet. Food intake measured by kcal/day was not significantly different among different dietary groups, but the HFD group gained more weight compared to the chow and HFC groups (Fig. 2A and B). The ratio of liver to body weight was increased nearly 2-fold in the LTKO mice as compared to the WT mice under the HFC dietary condition (*p* = 0.00045, Fig. 2C). Although there is a trend of increase in the HFD group, the ratio of perigonadal fat pad to body weight was not statistically different among three dietary groups (Fig. 2D).

### Diet and gene effects on serum triglyceride, cholesterol, and alanine aminotransferase (ALT)

**Figure 3:**
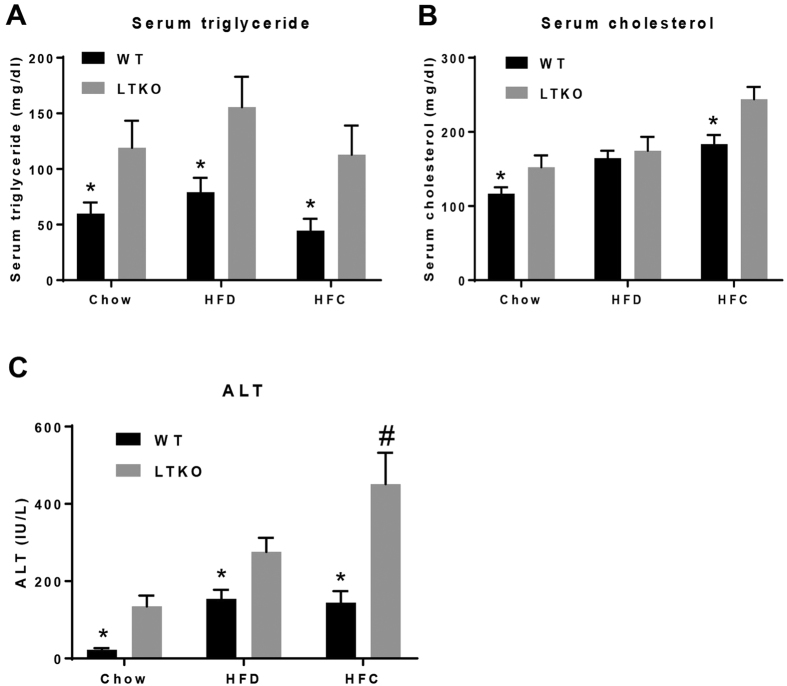
Blood chemistry analysis. (**A**–**C**) Serum triglyceride, cholesterol, and ALT in WT and LTKO mice that were fed with chow, HFD, or HFC diet, respectively. Values are expressed as mean ± SEM, n = 7 per group. *p < 0.05 for WT vs. LTKO, ^#^*p* < 0.05 for HFC vs. Chow or HFD in LTKO mice.

Serum triglyceride (TG) levels were significantly higher in the LTKO mice compared to the WT mice among all three dietary groups (*p* = 0.018, *p* = 0.022, and *p* = 0.023, respectively), but there was no significant effect by diet within the same genotype (Fig. 3A). Serum cholesterol levels were also elevated in the LTKO mice relative to the WT mice for the chow and HFC groups (*p* = 0.045 and *p* = 0.009, respectively, Fig. 3B). Serum ALT is often used a marker for liver injury. Interestingly, serum ALT levels were significantly affected by both Foxo1/3/4 deficiency and diets (*p* = 0.003, *p* = 0.012, and *p* = 0.003 for LTKO vs. WT in chow, HFD, and HFC, respectively; *p* = 0.05, *p* < 0.0001, and *p* = 0.012 for LTKO mice chow vs. HFD, chow vs. HFC, and HFD vs. HFC, respectively). The LTKO mice fed with the HFC diet had the highest level of serum ALT, followed by those fed with the HFD and chow diets (Fig. 3C).

### Effects on hepatic steatosis and fibrosis

**Figure 4:**
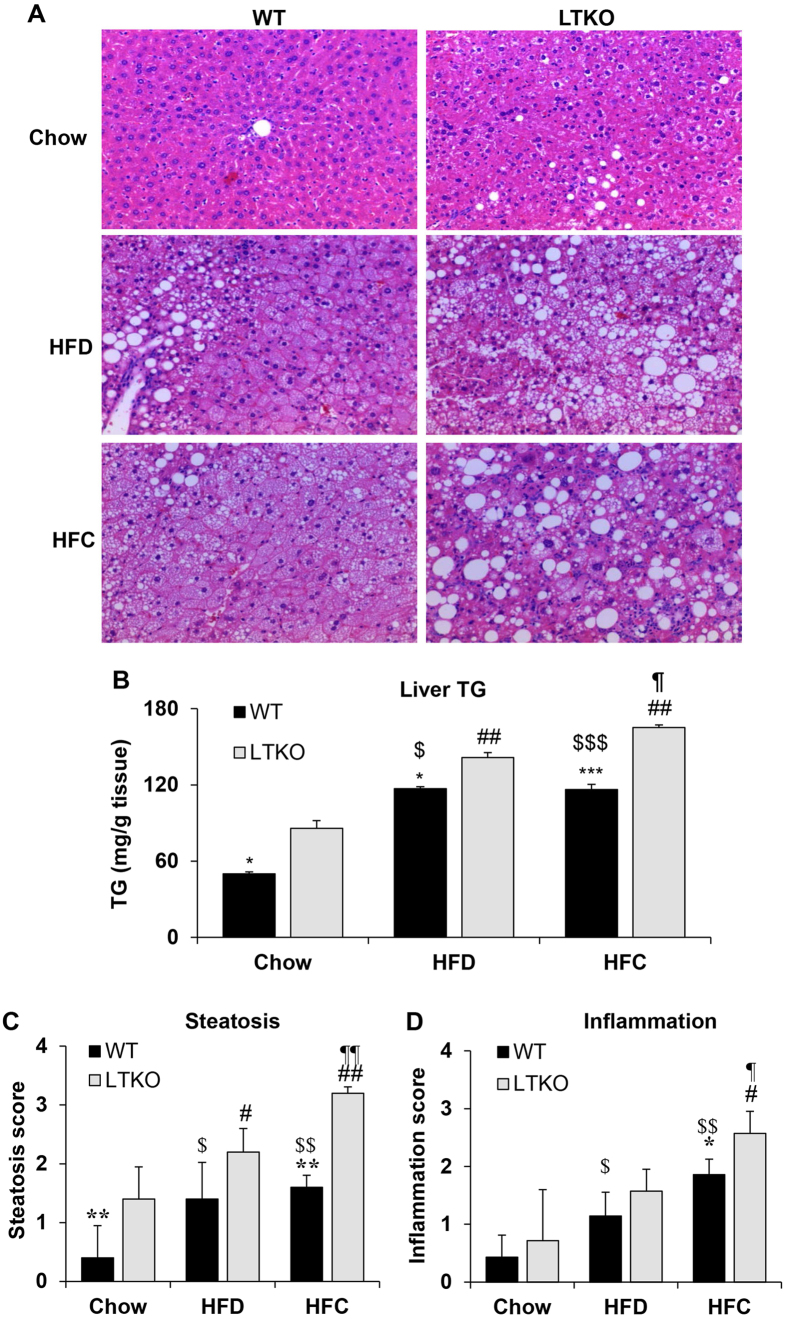
Hepatic steatosis and inflammation in WT and LTKO mice fed with different diets. (**A**) Liver sections were stained using H&E and imaged at a magnification of 200 ×. (**B**) Hepatic triglycerides was analyzed in the liver of WT and LTKO mice that were fed with chow, HFD, or HFC diet. Values are expressed as mean ± SEM, n = 3–4 for each group. *p < 0.05 and ***p < 0.001 for WT vs. LTKO, ^##^*p* < 0.01 for HFC or HFD vs. Chow in LTKO mice. ^$^*p* < 0.05 and ^$$$^*p* < 0.001 for HFC or HFD vs. Chow in WT. ^¶^*p* < 0.05 for HFC vs. HFD in LTKO. The scores of hepatic steatosis (**C**) and inflammation (**D**) were analyzed in WT and LTKO mice fed with chow, HFD, or HFC diet. Values are expressed as mean ± SEM, n = 4 for each group. *p < 0.05 and ***p < 0.001 for WT vs. LTKO, ^#^*p* < 0.05 and ^##^*p* < 0.01 for HFC or HFD vs. Chow in LTKO mice. ^$^*p* < 0.05 and ^$$^*p* < 0.01 for HFC or HFD vs. Chow in WT. ^¶^*p* < 0.05 and ^¶¶^*p* < 0.01 for HFC vs. HFD in LTKO.

**Figure 5:**
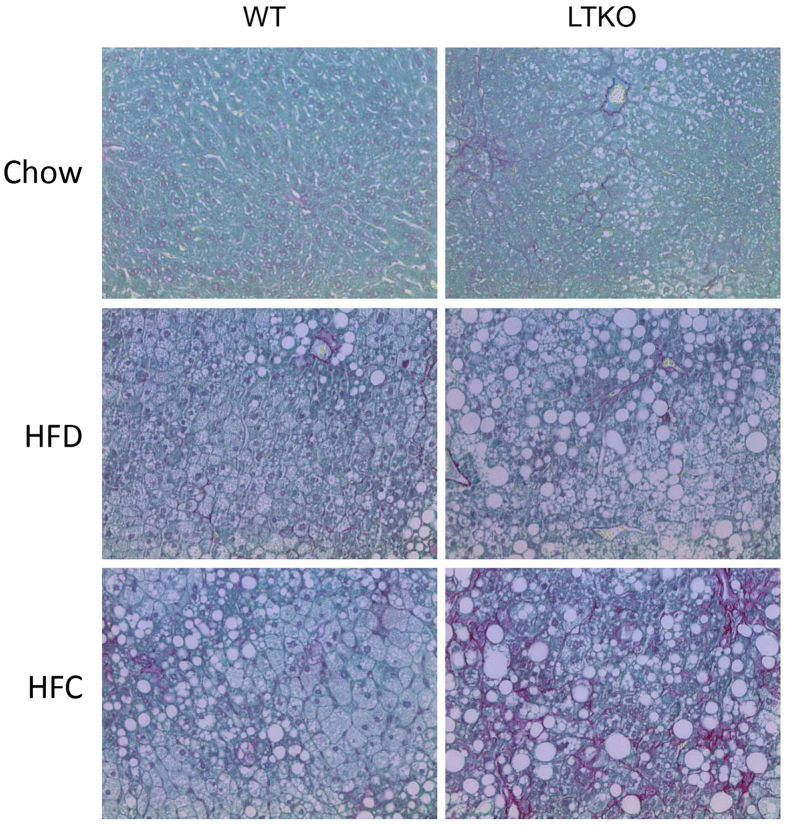
Fibrosis analysis of liver sections of WT and LTKO mice. Liver fibrosis was analyzed by Sirius Red staining (magnification 200X).

Liver sections were stained by hematoxylin and eosin for histological analysis. As shown in Fig. 4A, LTKO livers had increased lipid droplet accumulation in all three dietary conditions. However, liver tissues from mice fed with the HFC diet showed the worst hepatic steatosis with numerous large lipid droplets. Biochemical analysis of hepatic triglyceride contents also confirmed the steatosis phenotype in the LTKO mice under either HFD or HFC diet (Fig. 4B). Histological analysis also revealed increased hepatic steatosis and inflammation in the LTKO mice using the numerical scoring systems (Fig. 4C and D). Sirius Red stain was used to examine the presence of liver fibrosis. As shown in Fig. 5, fibrosis was increased in both HFD and HFC groups compared to the chow group. Foxo1/3/4 deficiency further exacerbated the extent of fibrosis in all dietary groups, especially in those fed with HFC, which showed a remarkable increase in fibrosis (Fig. 5).

### Effects on hepatic gene expression

**Figure 6:**
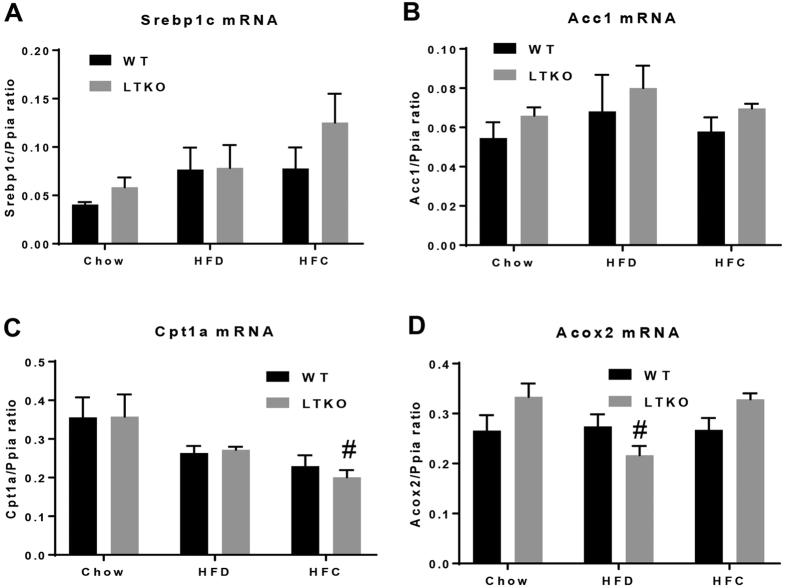
Expression of genes involving in lipid metabolism. (**A**–**D**) Srebp1c, Acc1, Cpt1a (^#^*p* < 0.05 for Chow-LTKO vs. HFC-LTKO), and Acox2 (^#^*p* < 0.05 for Chow-LTKO vs. HFD-LTKO) mRNA levels in WT and LTKO livers. Values are expressed as mean ± SEM, n = 4 per group.

**Figure 7:**
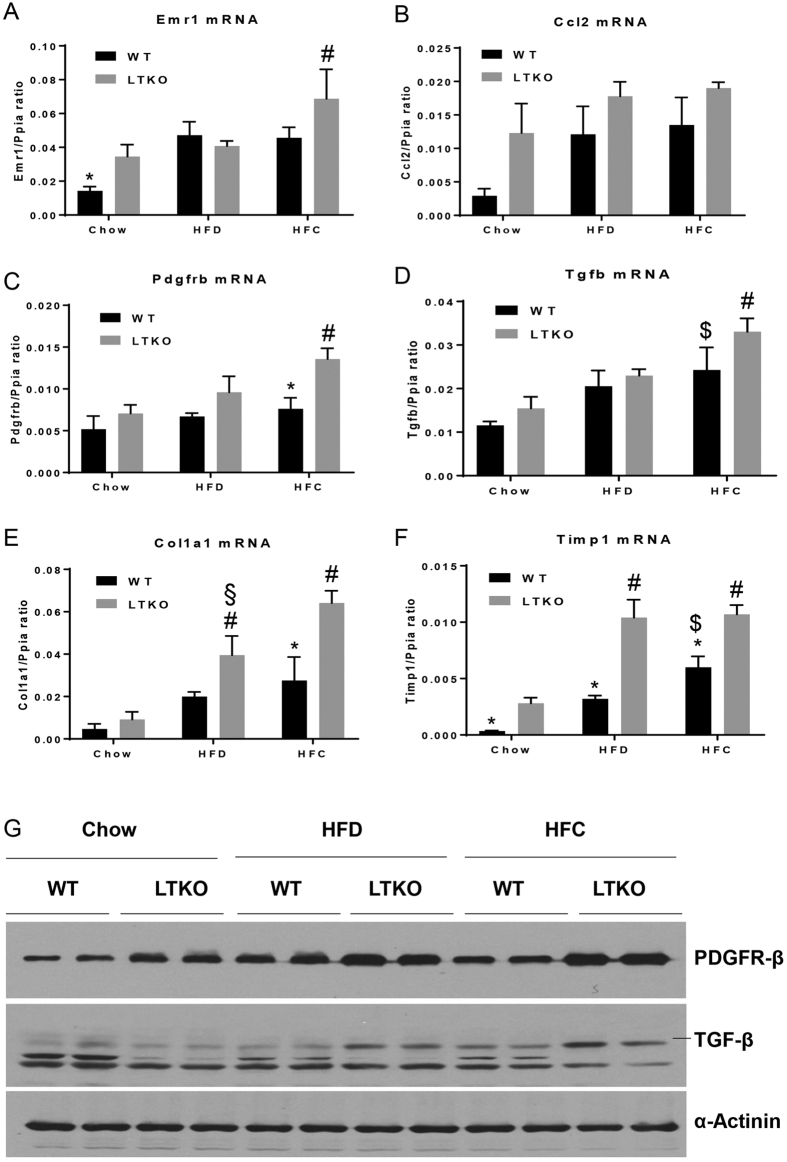
Expression of genes involving in inflammation and fibrogenesis. (**A**–**F**) Emr1 (*p < 0.05 for Chow-WT vs. Chow-LTKO, ^#^*p* < 0.05 for HFC-LTKO vs. Chow-LTKO), Ccl2, Pdgfrb (*p < 0.05 for HFC-WT vs. HFC-LTKO, ^#^*p* < 0.05 for HFC-LTKO vs. Chow-LTKO), Tgfb (^$^*p* < 0.05 for HFC-WT vs. Chow-WT, ^#^*p* < 0.05 for HFC-LTKO vs. Chow-LTKO), Col1a1 (*p < 0.05 for HFC-WT vs. HFC-LTKO, ^#^*p* < 0.05 for HFC- or HFD-LTKO vs. Chow-LTKO, ^§^*p* < 0.05 for HFD-LTKO vs. HFC-LTKO), and Timp1 (*p < 0.05 for LTKO vs. WT in the same diet group, ^#^*p* < 0.05 for HFD or HFC vs. Chow in the LTKO mice, ^$^*p* < 0.05 for HFC vs Chow in the WT mice) in WT and LTKO livers. Values are expressed as mean ± SEM, n = 4 per group. (**G**) Western blot analysis of PDGFRβ and TGFβ in the livers of WT and LTKO mice. The blots corresponding to the presented proteins are in the cropped format, and the full-length blots are presented in the Supplementary Figure 2.

To assess the molecular changes that lead to the phenotypic differences among the three dietary groups, we performed real-time PCR and Western blot analyses to determine differential expression of genes known to be involved in lipid metabolism and fibrogenesis. First, we examined the lipogenic genes including a key regulator *Srebp1c* and acetyl-CoA carboxylase 1 (*Acc1*). Although Srebp1c mRNA levels were increased in the liver of the HFC-treated mice, but they did not reach a statistical significance as compared to those fed with chow and HFD (Fig. 6A). Second, we analyzed genes that are involved in fatty acid oxidation – carnitine palmitoyltransferase 1a (*Cpt1a*) and acyl-CoA oxidase 2 (*Acox2*). Expression of Cpt1a was significantly decreased in the liver of the HFC-treated mice whereas Acox2 mRNA levels were decreased in the liver of the HFD-treated mice as compared to the Chow-WT mice (Fig. 6C and D). Third, as inflammation is a critical factor in the progression of fatty liver disease from simple steatosis to NASH, we also analyzed two inflammatory marker genes - adhesion G protein-coupled receptor E1 (*Adgre1*, also named Emr1 or F4/80) and chemokine (C-C motif) ligand 2 (*Ccl2*, also named MCP-1). Expression of the Emr1 gene in the WT livers was induced by either HFD or HFC diet whereas it was further elevated in the HFC-treated LTKO mouse livers (Fig. 7A). The hepatic Ccl2 mRNA levels were elevated in the LTKO mice compared to the WT mice under the chow diet condition but they did not reach a statistical significance. Fourth, we analyzed a few genes that are involved in the pathogenesis of hepatic fibrosis. Expression of platelet derived growth factor receptor, beta polypeptide (*Pdgfrb*) was elevated in the LTKO livers and further induced by the HFC diet (Fig. 7C). The HFC diet also significantly induced expression of the transforming growth factor beta gene (*Tgfb*) in both WT and LTKO livers (Fig. 7D). Hepatic expression of the type 1, alpha 1 collagen gene (*Col1a1*) was induced by either HFD or HFC, and Foxo1/3/4 deficiency further enhanced the Col1a1 gene expression (Fig. 7E). Like the Col1a1 gene, expression of the tissue inhibitor of metalloproteinase 1 gene (*Timp1*) was also induced by HFD or HFC and the Foxo1/3/4 gene knockout (Fig. 7F). The upregulation of the *Pdgfrb* and *Tgfb* genes was also confirmed at the protein levels (Fig. 7G).

**Figure 8:**
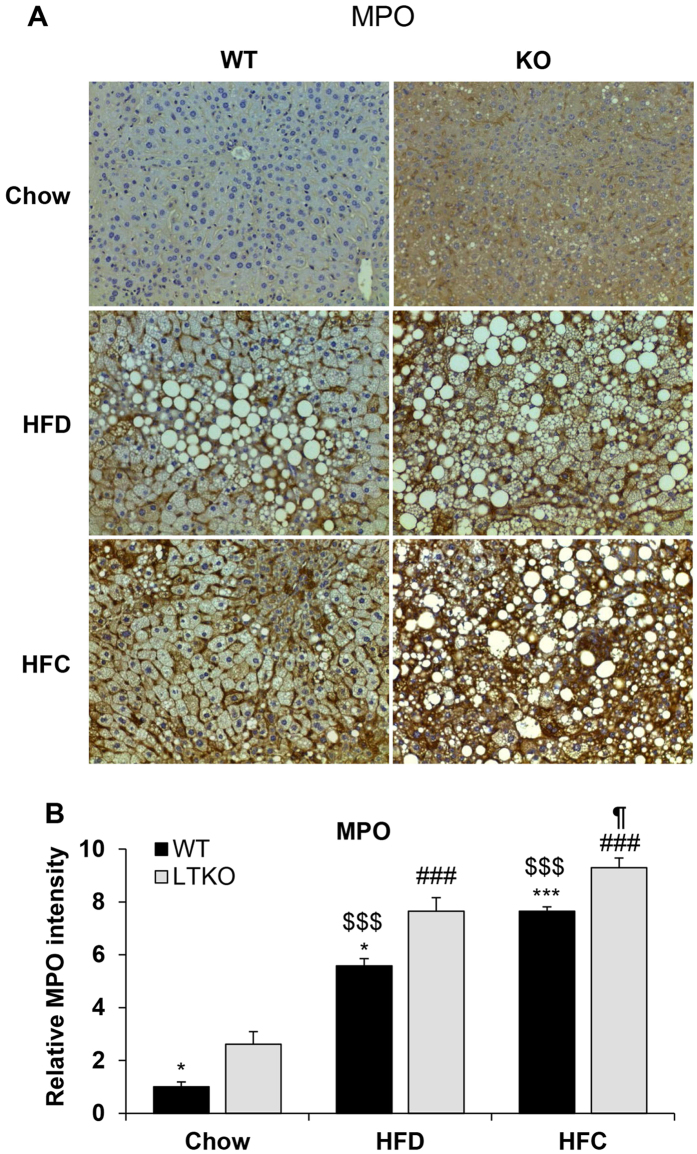
Hepatic neutrophil infiltration. (**A**) Hepatic inflammation was analyzed by IHC using MPO as a marker for neutrophils. The images were captured at a magnification of 200X. (**B**) The intensity of the MPO-positive staining was quantified and normalized to the Chow-WT group. The data are expressed as the means ± SEM (n = 4). *p < 0.05 and ***p < 0.001 for WT vs. LTKO, ^###^*p* < 0.001 for HFC or HFD vs. Chow in LTKO mice. ^$$$^*p* < 0.001 for HFC or HFD vs. Chow in WT. ^¶^*p* < 0.05 for HFC vs. HFD in LTKO mice.

**Figure 9:**
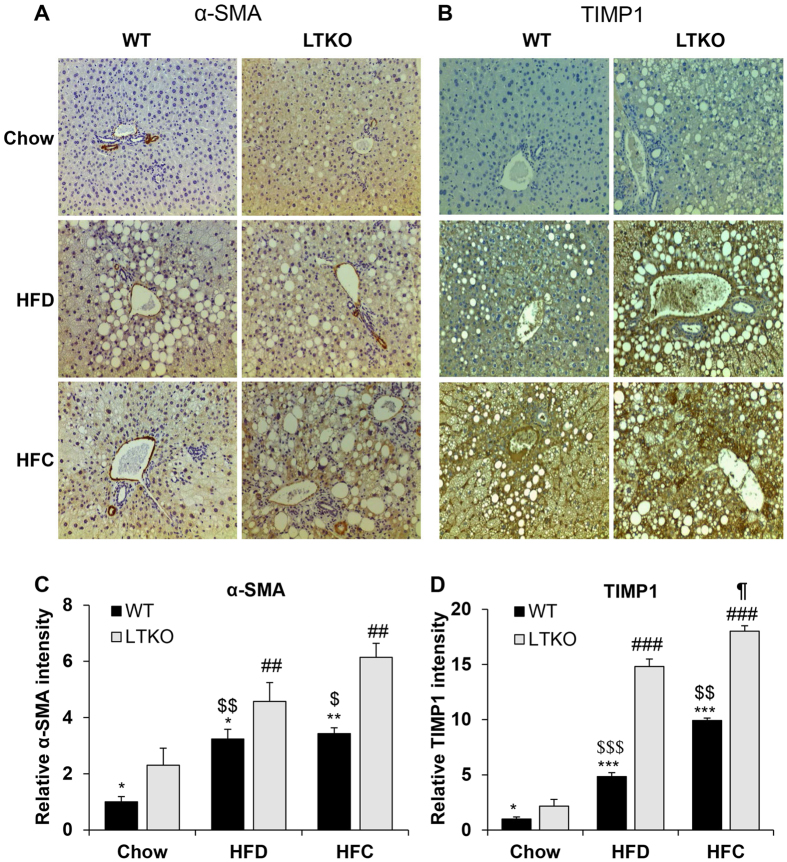
Expression of fibrotic markers in the liver. (**A** and **B**) Hepatic fibrosis was analyzed by IHC staining α-SMA and TIMP-1, respectively. All images were obtained at a magnification of 200X. (**C** and **D**) The intensity of the α-SMA and TIMP-1-positive staining was quantified and normalized to the Chow-WT group. The data are expressed as the means ± SEM (n = 4). *p < 0.05, **p < 0.01, and ***p < 0.001 for WT vs. LTKO, ^##^*p* < 0.01 and ^###^*p* < 0.001 for HFC or HFD vs. Chow in LTKO mice. ^$^*p* < 0.05, ^$$^*p* < 0.01, and ^$$$^*p* < 0.001 for HFC or HFD vs. Chow in WT. ^¶^*p* < 0.05 for HFC vs. HFD in LTKO mice.

Hepatic neutrophil infiltration is one of the hallmark features in NASH. We performed immunohistochemistry (IHC) analysis of liver sections using a common neutrophil marker – myeloperoxidase (MPO). Both HFD and HFC increased the neutrophil infiltration in WT and LTKO mouse livers, but the LTKO mice exhibited more severe inflammation evidenced by the increased MPO staining intensity (Fig. 8A and B). Since NASH also manifests hepatic fibrosis, we also analyzed two fibrosis markers — alpha-smooth muscle actin (α-SMA) and TIMP1, in liver sections. In comparison to WT mice, LTKO mice on both HFD and HFC diets showed more pronounced fibrosis demonstrated by the elevated α-SMA and TIMP1 staining intensity (Fig. 9A–D).

## Discussion

In this work, we have demonstrated two critical factors that significantly contribute to the pathogenesis of NAFLD. The first factor is intrinsic – the function of FOXO transcription factors. FOXOs, especially FOXO3, have implicated in animal longevity and metabolic homeostasis^38^. Our data from the Foxo1/3/4-LTKO mice indicate that deletion of *FOXO1/3/4* genes exacerbates diet-induced hepatic steatosis and liver injury, suggesting a protective role of FOXOs in the liver. The second factor is extrinsic or environmental – the diet composition. Not simply high content of saturated fat but moderately high content of fat plus cholesterol poses a higher risk for NAFLD.

From the biological functions of FOXOs, we are not surprised to see that FOXOs have protective effects on the development of NAFLD. There are several factors that contribute to the pathogenesis of steatosis: increased lipogenesis, increased uptake of free fatty acids, or decreased triglyceride breakdown or secretion in a form of VLDL^39^. Data from our laboratory and others have shown that FOXOs play a critical role in hepatic lipid homeostasis by inhibiting lipogenesis and increasing triglyceride breakdown^3^,^4^,^5^,^8^,^14^,^17^,^19^,^26^,^40^. In this work, we have observed that the expression of lipogenic and fatty acid oxidation genes is modulated by both FOXOs and diets. For example, the *Cpt1a* gene is significantly downregulated in HFC-treated LTKO livers whereas the *Acox2* gene was decreased in the HFD-treated LTKO livers. In addition, FOXO1 and FOXO6 have been shown to promote VLDL production in the liver by activating the expression of the microsomal triglyceride transfer protein^41^,^42^. Whether VLDL production is altered in the LTKO liver requires further investigation.

In recent years, cholesterol has been increasingly appreciated as a significant risk factor for NAFLD. A retrospective epidemiological study reveals that the ratio of total cholesterol to HDL-cholesterol is a significant risk factor for advanced NAFLD^31^. The association of cholesterol with NAFLD has also been reported in other studies^33^,^43^,^44^,^45^. In one study, C57BL/6 J mice fed with 45% fat (by calorie) plus 0.2% cholesterol diet for 4 months manifest phenotypic features of NASH and fibrosis^46^. In another study, Sprague-Dawley rats were fed either a high-fat diet (30% palm oil), high-fat (28.75% palm oil) plus 1.25% cholesterol diet, or high-fat (27.5% palm oil) plus 2.5% cholesterol diet for 9 weeks^34^. The results are very similar to our data that the cholesterol-containing diets remarkably promote progression from simple steatosis to NASH. More significantly, the 2.5% cholesterol-containing diet induces liver cirrhosis in 40% of rats^34^. However, the underlying mechanisms on high cholesterol diet causing NASH are not well understood. Excess hepatic free cholesterol is believed to have adverse effects on hepatocytes, Kupffer cells and hepatic stellate cells, including alteration of cell membrane fluidity and membrane protein function, endoplasmic reticulum stress, mitochondrial dysfunction, and activation of Kupffer and stellate cells^32^,^47^,^48^,^49^,^50^,^51^. Knockout of Foxo1/3 or Foxo1/3/4 in the mouse liver leads to elevated hepatic free cholesterol levels^7^,^40^, suggesting that FOXOs play a critical role in hepatic cholesterol homeostasis. As demonstrated in this work, deletion of hepatic Foxo1/3/4 markedly aggravates the NAFLD phenotype by increasing hepatic inflammation and fibrosis, especially on the HFC diet.

Although we have attempted to address both gene and dietary effects on the development of NAFLD, the diet-gene interplay is much more complicated than what we have presented here. First of all, diets have global effects on the animal system, including key organs involved in the metabolic homeostasis like brain, pancreas, intestine, muscle, and adipose tissue^52^,^53^. Second, both central and peripheral organs can impact liver physiology through inter-organ crosstalks^53^. For instance, white adipose tissue has strong interaction with hepatic lipid metabolism^53^,^54^,^55^. How those systemic and tissue-secreted factors may influence hepatic FOXO activity will be very interesting to be investigated in the future study.

In summary, our data reveals an important role of FOXO transcription factors in hepatic lipid homeostasis and their protection against the diet-induced fatty liver disease. These findings also suggest that the FOXO pathway may serve as a potential therapeutic target for NAFLD.

## Materials and Methods

### Animals and diets

All animal care and experimental procedures performed in this study were approved by the Institutional Animal Care and Use Committee of Indiana University School of Medicine in accordance with National Institutes of Health guidelines for the care and use of laboratory animals. Foxo1/3/4 floxed (as WT control) and LTKO mice were generated as previously described^8^. Both males and females were used in the experiments. At 2–3 months of age, animals were fed either regular chow (Teklad Diets 2018SX: 24% calories from protein, 18% calories from fat, and 58% calories from carbohydrate), high-fat diet (HFD, Research Diets D12492: 20% calories from protein, 60% calories from fat, and 20% calories from carbohydrate), or moderately high-fat-cholesterol diet (HFC, Research Diets D12109C, 20% calories from protein, 40% calories from fat, 40% calories from carbohydrate, and 1.25% cholesterol by weight). The feeding experiment was conducted for 3 months. In the end, the animals were sacrificed for blood and tissue collection. As males and females had a similar phenotype, the data presented in this report were primarily collected from the male mice.

### Blood chemistry and hepatic lipid analysis

Serum levels of cholesterol, triglycerides, and alanine aminotransferase were analyzed using commercial assay kits (Wako USA and Pointe Scientific, respectively) according to the manufacture’s manuals. Hepatic triglycerides were extracted and analyzed as previously described^8^.

### RNA isolation and real-time PCR analysis

Total RNAs from the liver tissues were isolated using TRI reagent (Sigma) as previously described^26^. cDNA was synthesized using a reverse transcription kit (Invitrogen). Real-time PCR analysis was performed using a SYBR Green PCR kit (Promega). Primer sequences are listed in the Supplementary Table 1.

### Protein analysis

Liver protein samples were prepared and analyzed as previously described^26^. The antibodies for the Foxo1, Foxo3, PDGF receptor β, and TGFβ proteins were purchased from Cell Signaling Technology (Beverly, MA), and the α-actinin and β-actin antibodies were purchased from Santa Cruz Biotechnology (Dallas, TX). The blots were scanned into digital files using a photo scanner (Epson V500).

### Histology and IHC analysis

Liver tissue samples were fixed in 10% formalin and processed for embedding and sectioning at the Histology Core of Indiana University School of Medicine. Liver sections (5 μm thickness) were stained with hematoxylin and eosin (H&E) or Sirius Red stain (Sigma). Immunohistochemistry analysis was performed for MPO, TIMP-1, and α-SMA. The liver tissue sections were deparaffinized, hydrated, and heated in 1 mM EDTA buffer for antigen retrieval at 100 °C for 5 min, and then treated with normal horse serum for 1 hour. Next, the slides were incubated with antibodies against MPO (1:100, Biocare Medical, Concord, CA), TIMP-1 (1:150, Proteintech, Rosemont, IL), or α-SMA (1:100, Cell Signaling Technology, Beverly, MA), for overnight. After washing with PBS buffer containing 0.05% Tween-20, the tissue sections were incubated with a biotinylated universal pan-specific antibody (PK-7200, Vectastain ABC kit, Vector Laboratories, Burlingame, CA) for 2 hours. The tissues were subsequently exposed to an avidin-biotin peroxidase complex (Vector Laboratories) for 1 hour. The peroxidase activity was visualized using a stable diaminobenzidine solution (Vector Laboratories). The images were captured using a regular microscope (200X magnification, Leica, Germany).

### Quantitative scoring analysis of steatosis and inflammation in liver sections

The quantitative scoring of H&E-stain based liver tissue sections was conducted according to the previous methods with slight modifications, regarding steatosis^56^,^57^ and inflammation^58^, respectively. Briefly, each section was examined by a specialist who was blinded to the sample information and hepatic steatosis and inflammation scores were evaluated. The degree of steatosis was graded ‘0’ to ‘4’ based on the average percent of fat-accumulated hepatocyte per field at 200 × magnification under H&E staining (grading 0, <5% of fat accumulation; grading 1, 5 to 25% of fat accumulation; grading 2, 26 to 50% of fat accumulation; grading 3, 51 to 75% of fat accumulation; grading 4, >75% of fat accumulation). The histopathological alterations for inflammation were also scored on 200X magnification H&E stained liver sections (grading 0, from normal, absence of pathology to <5% of maximum pathology; grading 1, <10% of maximum pathology: grading 2, 10% to 20% of maximum pathology; grading 3, >20% of maximum pathology).

The intensity of MPO, α-SMA and TIMP-positive signals in IHC images was quantified from the randomly selected sections at least five fields of each sample using Image J 1.46 software (NIH, Bethesda, MD). The staining intensity was normalized to the Chow-WT group and was presented as fold change.

### Statistical analysis

Data were presented as mean ± standard error (SEM). Comparisons between two groups were performed using two-tailed student t-test, and comparisons among multiple groups were analyzed using two-way ANOVA followed by Tukey post hoc test (GraphPad, La Jolla, CA). Differences with a *p* value < 0.05 were considered statistically significant.

## Additional Information

**How to cite this article**: Pan, X. *et al*. FOXO transcription factors protect against the diet-induced fatty liver disease. *Sci. Rep.* **7**, 44597; doi: 10.1038/srep44597 (2017).

**Publisher's note:** Springer Nature remains neutral with regard to jurisdictional claims in published maps and institutional affiliations.

## Supplementary Material

Supplementary Information
