# Supplementary material for: FOXO transcription factors protect against the diet-induced fatty liver disease
Source: Sci Rep. 2017 Mar 16;7:44597. doi: 10.1038/srep44597 (PMC5353679; doi:10.1038/srep44597)
Supplement: Supplementary Information [file srep44597-s1.pdf]

FOXO transcription factors protect against the diet-induced fatty liver disease

Xiaoyan Pan[1,2,#], Yang Zhang[2,#], Hyeong-Geug Kim[2], Suthat Liangpunsakul[3,4], X. Charlie Dong[2,*]

Supplementary Table 1
PCR primers

Name	DNA sequence (5′ to 3′)
Foxo1-F	ACGAGTGGATGGTGAAGAGC
Foxo1-R	TGCTGTGAAGGGACAGATTG
Foxo3-F	CTTCAAGGATAAGGGCGACA
Foxo3-R	GTTGATGATCCACCAAGAGC
Foxo4-F	GGTGCCCTACTTCAAGGACA
Foxo4-R	CCTTGATGAACTTGCTGTGC
Srebp-1c-F	GGAGCCATGGATTGCACATT
Srebp-1c-R	CCTGTCTCACCCCCAGCATA
Acc1-F	GGCCAGTGCTATGCTGAGAT
Acc1-R	TATCACACAGCCAGGGTCAA
Cpt1a-F	CCAGGCTACAGTGGGACATT
Cpt1a-R	AAGGAATGCAGGTCCACATC
Acox2-F	GAACATGCTCAGTCGCTTTG
Acox2-R	TGTCACCAACATGCCAAGAT
Emr1-F	TGCCTCCCTGACTTTCAAAT
Emr1-R	TGCCTCCCTGACTTTCAAAT
Ccl2-F	CCCAATGAGTAGGCTGGAGA
Ccl2-R	TCTGGACCCATTCCTTCTTG
Pdgfrb-F	CATTTGCAAAACCACCATTG
Pdgfrb-R	GGCATTCACAGAGACGTTGA
Tgfb-F	CGCAACAACGCCATCTATGA
Tgfb-R	ACTGCTTCCCGAATGTCTGA
Col1a1-F	CACCTGGTCCACAAGGTTTC
Col1a1-R	CCCATCATCTCCATTCTTGC
Timp1-F	CATGGAAAGCCTCTGTGGAT
Timp1-R	CTCAGAGTACGCCAGGGAAC

Supplementary Legends

Supplementary Figure 1. The full-length blots for the main Fig. 1D.

Supplementary Figure 2. The full-length blots for the main Fig. 7G.

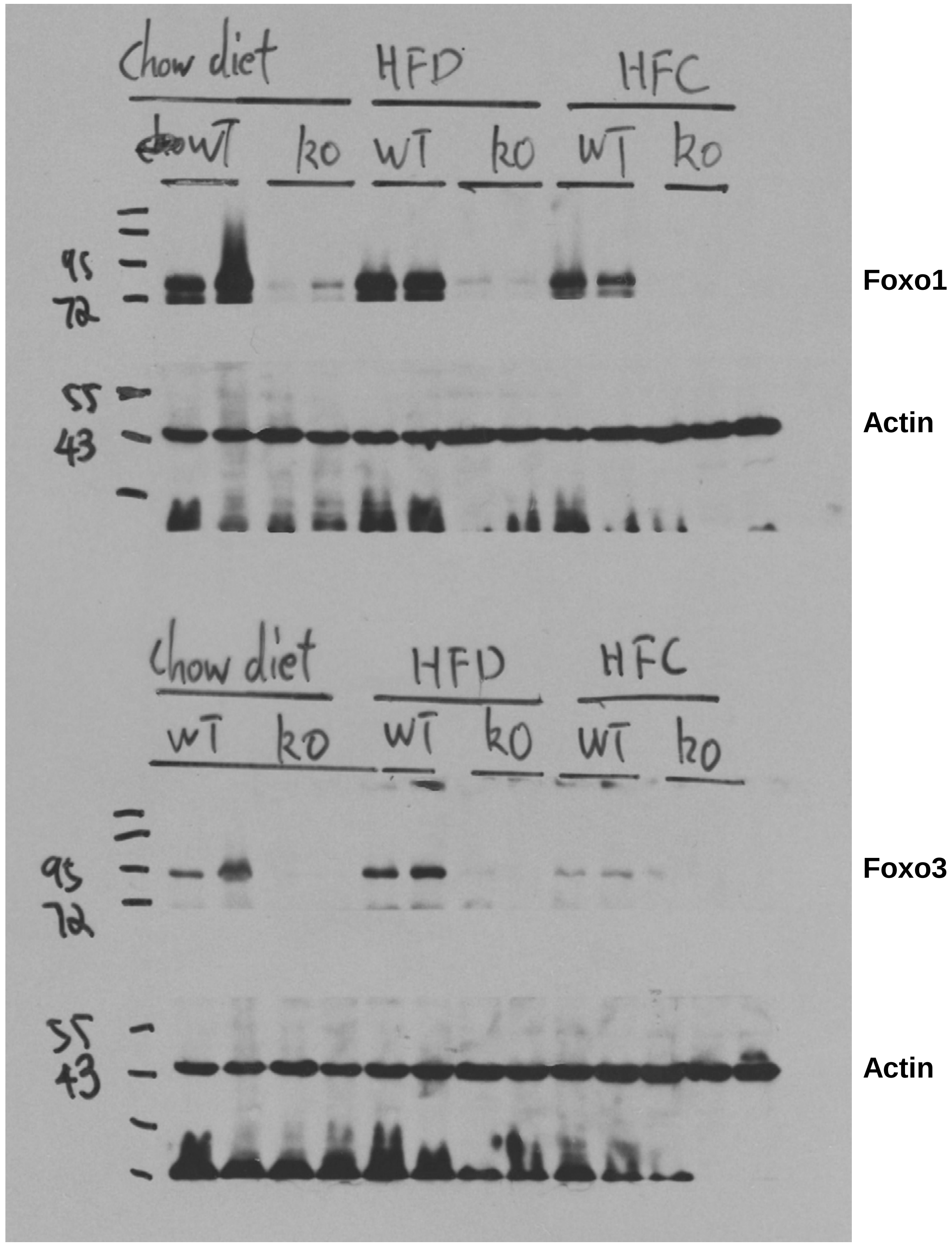
Chow diet
HFD
HFC
WT
ko
95
72
55
43
Foxo1
Actin
Foxo3

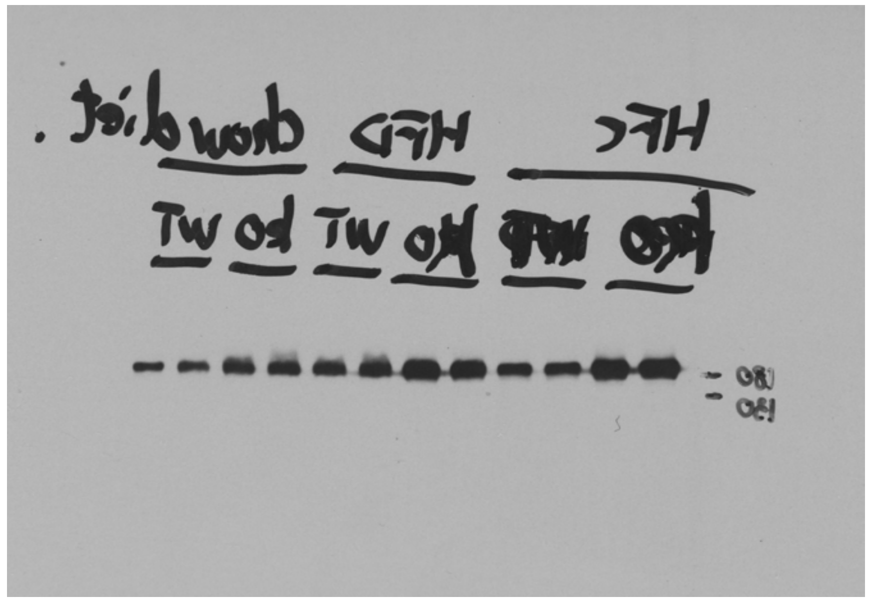

PDGRR-β

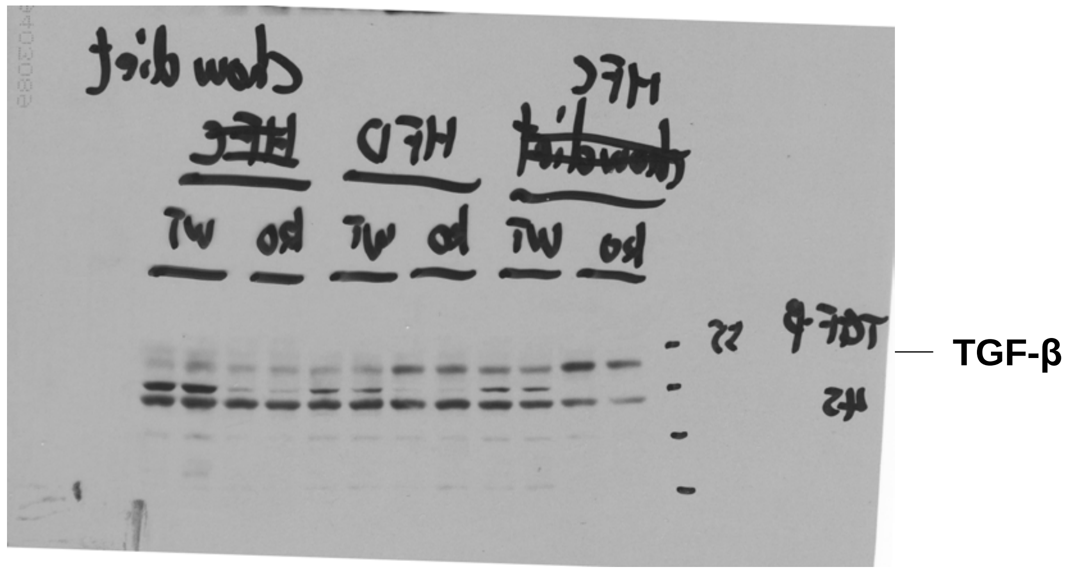

TGF-β

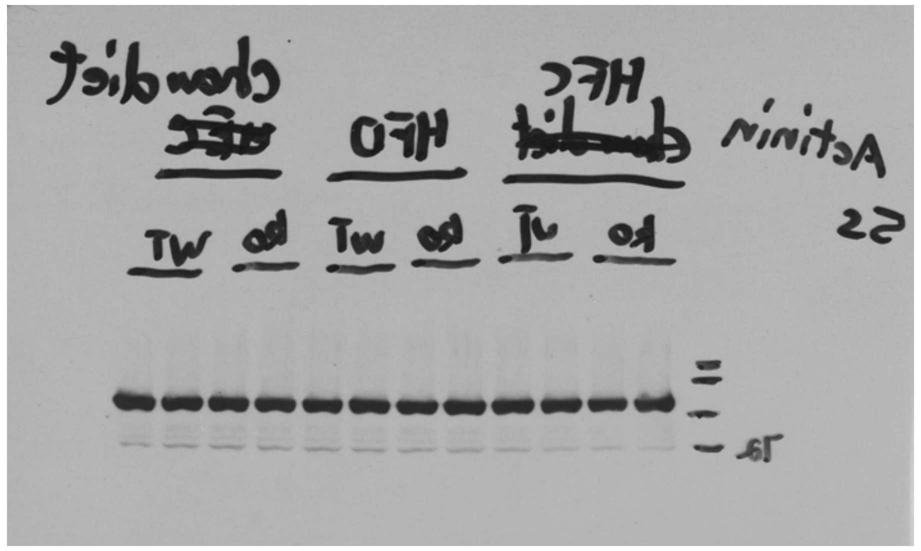

Actinin
